# Selective Serotonin Reuptake Inhibitor-Associated Bruxism: Neuropharmacologic Mechanisms and Clinical Implications

**DOI:** 10.7759/cureus.117729

**Published:** 2026-10-10

**Authors:** Binh Pham

**Affiliations:** 1 Sleep Medicine, Southwest Healthcare Medical Education Consortium, Temecula, USA

**Keywords:** 5-ht1a receptor, basal ganglia, bruxism, buspirone, dopamine, drug-induced movement disorders, neuropharmacology, selective serotonin reuptake inhibitors, serotonin, sleep bruxism

## Abstract

Selective serotonin reuptake inhibitor (SSRI)-associated bruxism is an increasingly recognized medication-associated phenomenon, although its incidence, neuropharmacologic mechanisms, and optimal management remain incompletely understood. This focused narrative review examines the clinical evidence, proposed mechanisms, and therapeutic implications of SSRI-associated bruxism. Available literature demonstrates recurring temporal associations between SSRI initiation or dose escalation and the emergence of jaw clenching, tooth grinding, pain, and related orofacial symptoms across multiple agents. Proposed mechanisms are multifactorial and may involve altered monoaminergic regulation within central motor networks, including serotonergic-dopaminergic interactions, 5-hydroxytryptamine (serotonin) receptor 1A (5-HT1A) signaling, and possible noradrenergic contributions; however, these mechanisms remain unconfirmed in affected patients. Adjunctive buspirone is the most consistently described pharmacologic strategy and may permit the continuation of an otherwise effective SSRI, although evidence for benefit remains primarily case-based. The literature is limited by heterogeneous bruxism definitions, inconsistent differentiation between awake and sleep manifestations, variable antidepressant exposures, and sparse objective physiologic assessment. Prospective studies using standardized assessment methods and controlled treatment designs are needed to clarify incidence, susceptibility, pathophysiology, and treatment response.

## Introduction and background

Bruxism is a repetitive jaw muscle activity characterized by the clenching or grinding of the teeth and/or by the bracing or thrusting of the mandible [1,2]. Sleep bruxism occurs without conscious voluntary control, whereas the awareness of awake bruxism may vary, and the behavior should not be uniformly classified as either voluntary or involuntary [1,2]. Contemporary international consensus distinguishes awake bruxism from sleep bruxism, which occur during wakefulness and sleep, respectively, and differ in their physiologic context and methods of assessment [1,2]. Bruxism is currently conceptualized as a behavior rather than an inherently pathologic disorder, with its clinical significance determined by the presence and severity of associated consequences [1,2]. Persistent or excessive bruxism may be associated with adverse oral and musculoskeletal consequences, and its clinical significance depends on the presence and severity of such consequences [1,2].

The understanding of bruxism has shifted from earlier models emphasizing peripheral factors such as dental occlusion to a predominantly central model of motor regulation [3]. Neurobiological studies implicate distributed interactions among cortical motor regions, subcortical structures, brainstem nuclei, the trigeminal motor system, and neural circuits involved in rhythmic masticatory activity [4]. Sleep bruxism is particularly associated with rhythmic masticatory muscle activity during sleep [5]. More recent syntheses incorporating human and animal evidence further support the involvement of coordinated central motor networks in the generation and modulation of jaw activity [6]. These findings provide a biological framework for understanding how centrally acting medications may alter orofacial motor behavior, although mechanisms derived from spontaneous sleep bruxism should not automatically be generalized to medication-associated bruxism [7].

Central motor control is also modulated by monoaminergic neurotransmitter systems, including serotonin, dopamine, and norepinephrine, which influence neuronal excitability, motor output, and sensorimotor integration within cortical, subcortical, and brainstem networks [4,6,7]. Alterations in the balance among these systems therefore provide a biologically plausible basis for medication-associated disturbances in jaw motor activity [4,6,7]. However, the contribution of individual neurotransmitters, receptor subtypes, and neural circuits to drug-associated bruxism remains incompletely defined, and mechanistic hypotheses derived from general motor physiology should be distinguished from mechanisms demonstrated directly in affected patients.

Selective serotonin reuptake inhibitors (SSRIs) are antidepressants that inhibit the serotonin transporter, thereby reducing serotonin reuptake and increasing serotonergic signaling. SSRIs have also been repeatedly implicated in medication-associated bruxism [8]. In the general adult population, sleep bruxism has been reported in approximately 8% of individuals [7], whereas one cross-sectional study of antidepressant users reported bruxism in 24.3% of treated patients compared to 15.3% of controls [9]. These figures should be interpreted cautiously because the observational study was not restricted to SSRIs and relied on clinically reported rather than objectively confirmed bruxism. A systematic review of published cases further identified a recurring temporal relationship between serotonergic antidepressant exposure and the emergence of jaw clenching, tooth grinding, jaw pain, and related orofacial symptoms, frequently after treatment initiation or dose escalation [8]. Collectively, the available evidence supports a clinically meaningful association between SSRI exposure and bruxism while leaving the true incidence, comparative risk among individual agents, dose dependence, and patient-specific susceptibility incompletely defined.

One frequently proposed neuropharmacologic hypothesis is that SSRI-associated bruxism may involve altered serotonergic-dopaminergic interactions within central motor pathways [8,10]. Increased serotonergic neurotransmission has been hypothesized to modify the dopaminergic regulation of orofacial motor activity, while reported improvement with adjunctive buspirone provides an additional pharmacologic clue [10]. These observations remain indirect and should be interpreted as supporting a biologically plausible model rather than confirming a specific receptor abnormality, the restoration of dopaminergic neurotransmission, or a serotonin-dopamine imbalance as the sole mechanism of SSRI-associated bruxism [8,10].

Despite the growing recognition of SSRI-associated bruxism, an important gap remains between the clinical literature describing the adverse effect and the neuropharmacologic evidence proposed to explain it. Direct human mechanistic studies are lacking, and the available evidence is dispersed across case reports, observational studies, broader bruxism neurobiology, and pharmacologic literature [8,10]. Accordingly, this focused narrative review examines the clinical evidence and proposed neuropharmacologic mechanisms underlying SSRI-associated bruxism, with particular attention to central motor-control networks, serotonergic-dopaminergic interactions, 5-hydroxytryptamine (serotonin) receptor 1A (5-HT1A) receptor signaling, buspirone, and the potential contribution of additional monoaminergic systems. The review also considers the temporal relationship between SSRI exposure and symptom development, clinical manifestations, reported management strategies, and major limitations of the available evidence. Throughout the review, direct clinical findings are distinguished from mechanistic inference so that biological plausibility is not interpreted as established causality. The principal proposed mechanisms are summarized in Table 1.

**Table 1:** Proposed neuropharmacologic hypotheses potentially underlying SSRI-associated bruxism This table summarizes proposed neuropharmacologic hypotheses concerning the pathophysiology of SSRI-associated bruxism. The proposed pathways should be interpreted as biologically plausible hypotheses rather than established causal mechanisms. Evidence is derived primarily from broader bruxism neurobiology, clinical observations, case-based evidence, and pharmacologic inference. Clinical and pharmacologic evidence concerning buspirone as a potential management strategy is discussed separately from the mechanistic hypotheses summarized here 5-HT1A, 5-hydroxytryptamine (serotonin) receptor 1A; SSRI, selective serotonin reuptake inhibitor

Proposed hypothesis	Neuropharmacologic rationale	Representative supporting evidence	Current limitations
Serotonergic modulation of dopaminergic motor pathways	Increased serotonergic signaling during SSRI therapy may alter dopaminergic regulation within central motor pathways, potentially facilitating abnormal jaw motor activity [8,10].	Clinical temporal associations and pharmacologic observations have been interpreted as consistent with a possible serotonergic-dopaminergic interaction [8,10].	Direct evidence demonstrating reduced dopaminergic neurotransmission in affected patients is lacking, and the proposed interaction remains inferential [8,10].
5-HT1A receptor-mediated serotonergic modulation	Altered 5-HT1A signaling may modify serotonergic tone and downstream monoaminergic regulation within central motor networks [8,10].	Pharmacologic observations in the clinical literature have contributed to the hypothesis that 5-HT1A-mediated modulation may influence SSRI-associated bruxism [8,10].	No direct evidence demonstrates that 5-HT1A receptor dysfunction is present in affected patients or represents a primary pathogenic mechanism [8,10].
Brainstem and trigeminal motor circuit dysregulation	Altered monoaminergic modulation of the trigeminal motor system and rhythmic masticatory circuitry may influence motor excitability and jaw activity [3,4,6,7].	Broader bruxism neurobiology supports the central regulation of rhythmic masticatory activity through coordinated cortical, brainstem, and trigeminal motor networks [3,4,6,7].	Evidence is derived largely from general and sleep-bruxism neurobiology rather than the direct investigation of SSRI-associated bruxism [3,4,6,7].
Broader monoaminergic network dysregulation	SSRI-associated bruxism may reflect altered interactions among multiple monoaminergic systems rather than the dysfunction of a single neurotransmitter pathway [3,8].	Available neurobiological and clinical observations are compatible with a multifactorial model of central monoaminergic dysregulation [3,8].	The relative contribution of individual neurotransmitter systems remains uncertain, and no unified mechanistic model has been validated [3,8].

## Review

Literature search strategy

A focused narrative review of the literature was conducted in September 2026 to identify clinical evidence regarding SSRI-associated bruxism and the neurobiological and pharmacologic context relevant to proposed mechanisms. PubMed was searched through September 2026 without restriction on publication date, with searches limited to English-language publications. Google Scholar was used as a supplementary discovery and citation-tracking tool rather than as an exhaustive bibliographic database. Reference lists of relevant primary studies, systematic reviews, narrative reviews, and case reports were also manually examined to identify additional publications.

The principal PubMed search combined bruxism-related terms (“bruxism,” “sleep bruxism,” “awake bruxism,” or “jaw clenching”) with SSRI-related terms (“selective serotonin reuptake inhibitor,” “SSRI,” “serotonergic antidepressant,” “fluoxetine,” “sertraline,” “paroxetine,” “citalopram,” “escitalopram,” or “fluvoxamine”). Additional targeted searches combined bruxism-related terms with “buspirone,” “5-HT1A,” “serotonin,” “dopamine,” “norepinephrine,” “pharmacovigilance,” “basal ganglia,” “trigeminal motor system,” “masticatory central pattern generator,” and “drug-induced movement disorder.” Broader antidepressant-related literature was considered when relevant to the interpretation of SSRI-specific findings.

Clinical publications were considered relevant when they described bruxism, jaw clenching, tooth grinding, or related orofacial motor symptoms during SSRI exposure and provided clinically informative data regarding the implicated medication, the timing of symptom onset, dose changes, clinical manifestations, management, or outcomes. Case reports and case series were retained because they comprise a substantial portion of the available literature. Observational studies, systematic reviews, and pharmacovigilance analyses were also considered, with studies combining SSRIs with serotonin-norepinephrine reuptake inhibitors (SNRIs) or other antidepressant classes interpreted separately when SSRI-specific effects could not be isolated. Case reports and case series were interpreted primarily for temporal association, medication changes, dechallenge or rechallenge information, and treatment response, whereas observational and pharmacovigilance studies were used to provide broader associative evidence rather than establish individual-level causality or incidence.

Mechanistic evidence was evaluated separately from evidence supporting the clinical association. Neurobiological, pharmacologic, and motor-physiology studies were included when they provided relevant context regarding the central regulation of jaw activity, serotonergic-dopaminergic interactions, 5-HT1A signaling, buspirone pharmacology, noradrenergic modulation, or brainstem and trigeminal motor circuitry. Such evidence was used to assess biological plausibility rather than to establish a specific receptor-level or circuit-level mechanism in patients with SSRI-associated bruxism. Studies incorporating the objective assessment of bruxism were given greater interpretive weight than those relying exclusively on self-report or routine clinical observation, and awake and sleep bruxism were treated as related but distinct phenomena.

Because the available literature is heterogeneous in study design, population, antidepressant exposure, bruxism definition, assessment method, and outcome, quantitative pooling was not considered appropriate. Findings were therefore synthesized qualitatively according to study design, clinical relevance, and methodological limitations. This article was designed as a focused narrative review rather than a systematic review or meta-analysis; no formal risk-of-bias instrument was applied, and study selection did not follow predefined systematic-review procedures. Potential limitations related to language restriction, database selection, the supplementary use of Google Scholar, relevance-based study selection, and the heterogeneity of the underlying evidence are discussed further in the Strengths, limitations, and future directions section.

Neuropharmacologic mechanisms potentially underlying SSRI-associated bruxism

The neuropharmacologic basis of SSRI-associated bruxism remains incompletely understood and is likely multifactorial. Bruxism is increasingly viewed as a centrally regulated motor behavior involving coordinated activity across cortical and subcortical motor regions, brainstem nuclei, the trigeminal motor system, and circuitry responsible for rhythmic masticatory activity [3,4,6,7]. These networks are modulated by serotonergic, dopaminergic, and noradrenergic neurotransmission, providing a biologically plausible framework through which medications that alter monoaminergic signaling may disrupt orofacial motor control [3,4,6,7]. However, this framework is derived primarily from the broader neurobiology of bruxism, whereas the SSRI-specific literature remains predominantly clinical and case-based rather than directly mechanistic [8,10].

One frequently proposed neuropharmacologic hypothesis is that SSRI-associated bruxism may involve altered serotonergic-dopaminergic interactions within central motor pathways [8,10]. Enhanced 5-hydroxytryptamine (5-HT) neurotransmission during SSRI treatment has been hypothesized to reduce dopaminergic influence within motor regulatory networks, potentially producing a relative hypodopaminergic state that facilitates abnormal jaw motor activity [8,10]. This model provides a plausible connection between SSRI pharmacology and the emergence of bruxism and is consistent with reports of symptoms developing after treatment initiation or dose escalation and improving after the reduction or withdrawal of serotonergic exposure [8]. Nevertheless, direct evidence demonstrating reduced dopaminergic neurotransmission in affected patients is lacking. The serotonin-dopamine imbalance model should therefore be regarded as a mechanistic hypothesis rather than an established pathogenic pathway.

Clinical observations involving buspirone, a nonbenzodiazepine anxiolytic with partial agonist activity at 5-HT1A receptors, provide indirect pharmacologic evidence that SSRI-associated bruxism may be modifiable through monoaminergic mechanisms, but they do not identify the causal pathway. In the four-case series by Bostwick and Jaffee, all four patients had recently initiated sertraline and developed new-onset complaints attributed to bruxism; all four subsequently experienced relief of bruxism and associated symptoms after adjunctive buspirone was added [10]. A subsequent report involving fluoxetine described a similar clinical response [11]. These observations suggest that pharmacologic pathways affected by buspirone may modify the clinical phenotype, but case-based treatment responses do not establish treatment efficacy or identify a specific receptor-level mechanism [10,11].

Evidence from broader antidepressant pharmacology also argues against a purely serotonergic or single-receptor model. In a World Health Organization pharmacovigilance analysis, Montastruc identified bruxism reporting signals involving several serotonergic antidepressants and found that reporting was more closely associated with norepinephrine transporter affinity than with serotonin transporter affinity [12]. Because pharmacovigilance disproportionality analyses cannot establish incidence or causality, this finding should be interpreted cautiously. Nevertheless, it suggests that the magnitude of serotonin transporter inhibition alone may not explain antidepressant-associated bruxism and raises the possibility that noradrenergic and other monoaminergic mechanisms contribute. Broader postmarketing data have also identified antidepressant-associated reporting signals across a spectrum of movement disorders that include bruxism, akathisia, dystonia, parkinsonism, and tardive dyskinesia [13]. This broader movement-disorder context is compatible with overlapping monoaminergic motor mechanisms, although pharmacovigilance associations do not establish a shared pathophysiology.

Experimental buspirone pharmacology further supports caution in assigning a simple receptor-specific mechanism. Liu et al. demonstrated that the neurobehavioral and neurochemical effects of buspirone differed between acute and chronic administration and that selected effects were dependent on 5-HT1A receptor activation [14]. These findings support the biological plausibility of adaptive serotonergic mechanisms. Importantly, the study did not demonstrate a consistent increase in dopamine efflux and was not designed to investigate bruxism [14]. The clinical effect of buspirone should therefore not be attributed solely to presynaptic 5-HT1A autoreceptor activation or the restoration of dopaminergic neurotransmission. Rather, the available findings are more consistent with complex downstream monoaminergic modulation. Accordingly, improvement with buspirone does not demonstrate that 5-HT1A receptor dysfunction is present in affected patients, that such dysfunction causes SSRI-associated bruxism, or that the normalization of dopaminergic signaling mediates the therapeutic response [10,11,14].

At the receptor level, broader serotonergic-dopaminergic literature provides one possible explanation for the proposed reduction in dopaminergic signaling. Preclinical pharmacologic evidence indicates that 5-HT2C receptor signaling can inhibit tonic and evoked dopamine release, whereas the effects of 5-HT2A signaling are more heterogeneous and may facilitate dopamine release in some circuits, emphasizing that the serotonergic regulation of dopamine is receptor- and circuit-dependent [15]. A 5-HT2C-mediated inhibitory effect therefore represents one biologically plausible extension of the serotonin-dopamine hypothesis in SSRI-associated bruxism, but this pathway has not been demonstrated directly in affected patients.

Taken together, the available evidence is consistent with a broader hypothesis in which SSRI-associated bruxism may reflect altered monoaminergic regulation within distributed motor networks, with serotonergic-dopaminergic interactions, 5-HT1A-mediated modulation, possible 5-HT2C-mediated effects, and noradrenergic contributions representing complementary rather than mutually exclusive hypotheses [8,10-15]. Direct receptor-level or circuit-level confirmation in affected humans remains lacking; accordingly, these models should be interpreted as biologically plausible hypotheses rather than established mechanisms.

Clinical evidence of SSRI-associated bruxism

The evidence relevant to SSRI-associated bruxism includes both SSRI-specific clinical reports and broader studies of antidepressant-associated bruxism. Garrett and Hawley reviewed 46 published cases of antidepressant-associated bruxism, with SSRIs accounting for 74% of the implicated medications [8]. Within that broader antidepressant-associated case literature, fluoxetine and sertraline were among the most frequently reported agents, and symptoms commonly emerged within approximately three to four weeks of antidepressant initiation or dose titration [8]. Reported manifestations included teeth grinding, jaw clenching, jaw pain, and trismus, while improvement was frequently described following dose reduction, medication discontinuation, substitution, or adjunctive buspirone [8]. These findings provide clinically relevant support for an association involving SSRIs, but because the systematic review included antidepressants beyond the SSRI class and was based largely on published case reports, it cannot establish SSRI-specific incidence, comparative risk among individual SSRIs, or medication causality.

Observational studies provide broader population-level context but introduce different limitations. Uca et al. evaluated 807 participants and reported bruxism in 24.3% of antidepressant users compared to 15.3% of controls [9]. The authors additionally reported a 14.0% rate of antidepressant-attributed bruxism, which they termed incidence; however, because the study used a cross-sectional design rather than longitudinal follow-up, this estimate should not be interpreted as a conventional incidence rate [9]. The study evaluated antidepressants broadly rather than SSRIs alone, and sleep bruxism was identified from participant reports rather than objective physiologic assessment. Individual reports have nevertheless described similar temporal relationships with several SSRIs. Milanlioglu reported paroxetine-associated sleep bruxism that improved with adjunctive buspirone [16], Ak et al. described two additional cases associated with serotonin reuptake inhibitor treatment [17], and Kohat and Chenchula more recently reported sertraline-associated bruxism with improvement after medication modification and subsequent adjunctive buspirone [18]. Such reports provide useful challenge, dechallenge, and treatment-response observations but remain susceptible to confounding, selective reporting, and publication bias.

Instrument-based evidence is considerably more limited. Isa Kara et al. prospectively evaluated 30 patients initiating either fluoxetine or paroxetine using BiteStrip, a portable device that records masseter muscle activity, before the first SSRI dose and again on days 7 and 15 [19]. BiteStrip scores increased significantly after treatment, with increased scores observed in most participants by days 7 and 15, whereas only a minority of patients reported awareness of newly developed or worsened bruxism [19]. This divergence between instrument-recorded activity and patient awareness is clinically important because symptom reporting alone may underestimate changes in nocturnal jaw muscle activity [19]. BiteStrip provided portable masseter muscle activity assessment rather than full polysomnographic characterization, and the study was limited to fluoxetine and paroxetine over a brief observation period [19]. The findings therefore provide evidence of treatment-associated changes in jaw muscle activity but do not establish a uniform physiologic effect across the SSRI class [19].

More recent observational data further support an association while illustrating the difficulty of isolating SSRI-specific effects. Santos et al. retrospectively evaluated 583 patients attending a temporomandibular disorder clinic, of whom 53 were receiving an SSRI or serotonin-norepinephrine reuptake inhibitor [20]. Bruxism was identified in 43 of these 53 antidepressant users, and logistic regression demonstrated higher odds of bruxism among SSRI/SNRI users compared to nonusers (odds ratio, 2.52; 95% confidence interval, 1.21-5.23; p=0.014) [20]. The study also evaluated psychosocial factors, including stress, anxiety, and depression, which were not significantly associated with bruxism [20]. However, SSRIs and serotonin-norepinephrine reuptake inhibitors were analyzed together, the sample was derived from a specialty-clinic population, and bruxism was identified using self-report, together with clinical evidence of tooth wear rather than standardized prospective physiologic assessment. These findings therefore support an association between serotonergic antidepressant exposure and bruxism but do not provide an SSRI-specific risk estimate or establish medication causality.

Pharmacovigilance analyses provide a complementary perspective by identifying reporting signals across large international adverse-event databases [12,13]. Such analyses are useful for detecting medication-associated patterns that may be difficult to recognize in smaller clinical studies, but they cannot establish incidence or individual-level causality because of underreporting, stimulated reporting, incomplete clinical characterization, variable diagnostic accuracy, and the absence of reliable exposure denominator data [12,13]. In one pharmacovigilance analysis, bruxism reporting showed a stronger association with norepinephrine transporter affinity than with serotonin transporter affinity [12]. This observation should be regarded as exploratory and hypothesis-generating and should not be interpreted as evidence of a specific noradrenergic mechanism or as excluding a serotonergic contribution. Pharmacovigilance data therefore strengthen the recognition of the clinical signal while providing limited certainty regarding the underlying neuropharmacologic mechanism.

The principal weakness of the clinical literature is the heterogeneity of how bruxism has been defined and assessed. Leonan-Silva et al. critically appraised evidence encompassing 41 patients described in case reports, 17 patients in case series, and 2,655 participants in cross-sectional studies [21]. Most evidence relied on self-reported or clinically reported bruxism, polysomnography was used in only one study, and awake and sleep bruxism were inconsistently differentiated [21]. No study prospectively evaluated bruxism before and after antidepressant initiation while adequately controlling for relevant psychological confounding variables [21]. Differences in pharmacodynamic and pharmacokinetic properties among individual SSRIs could theoretically contribute to variation in susceptibility or clinical expression; however, the available literature does not provide sufficiently robust comparative evidence to determine whether such differences meaningfully alter bruxism risk across individual agents [8,12,21]. These limitations are important because subjective jaw clenching, self-reported tooth grinding, clinically inferred bruxism, instrument-recorded jaw muscle activity, and polysomnographically characterized sleep bruxism represent related but noninterchangeable outcomes.

Taken together, the available clinical literature supports a reproducible association between serotonergic antidepressant exposure and the emergence or worsening of bruxism, particularly when symptoms develop after treatment initiation or dose escalation and improve following medication modification [8,16-21]. The recurrence of similar temporal patterns across several SSRIs, together with observational, instrument-based, and pharmacovigilance findings, strengthens the clinical signal beyond isolated case reports. However, the evidence remains insufficient to establish true SSRI-specific incidence, comparative risk among individual agents, a consistent dose-response relationship, or a uniform physiologic phenotype. The clinical association is therefore better supported than any specific causal or neuropharmacologic mechanism. The principal clinical evidence, study characteristics, key findings, and major methodological limitations are summarized in Table 2.

**Table 2:** Representative clinical evidence concerning SSRI-associated bruxism This table summarizes representative clinical evidence concerning the association between serotonergic antidepressant exposure and bruxism. The evidence includes SSRI-specific studies, as well as broader antidepressant, mixed SSRI/SNRI, and pharmacovigilance data; these sources should therefore not be interpreted as providing equivalent SSRI-specific evidence OR, odds ratio; SNRI, serotonin-norepinephrine reuptake inhibitor; SSRI, selective serotonin reuptake inhibitor; CI, confidence interval

Evidence source	Study design/population	Principal findings	Key limitations
Garrett and Hawley [8]	Systematic review of 46 published cases of antidepressant-associated bruxism	SSRIs accounted for 74% of implicated medications. Fluoxetine and sertraline were among the most frequently reported agents, and symptoms commonly emerged within approximately three to four weeks of antidepressant initiation or dose titration.	Evidence was predominantly case-based and cannot establish incidence, comparative risk among individual SSRIs, or causality.
Uca et al. [9]	Observational study of 807 participants	Bruxism was reported in 24.3% of antidepressant users compared to 15.3% of controls; the authors also reported a 14.0% rate of antidepressant-attributed bruxism, which they termed incidence.	Antidepressants were evaluated broadly rather than SSRIs alone, and sleep bruxism was based on participant report rather than objective physiologic assessment.
Milanlioglu [16]; Ak et al. [17]; Kohat and Chenchula [18]	Individual case reports and small case-based observations	Similar temporal relationships were described with several serotonergic antidepressants, including the emergence of bruxism during treatment and improvement following medication modification.	Small samples, potential confounding, selective reporting, and publication bias limit causal inference.
Isa Kara et al. [19]	Prospective study of 30 patients initiating fluoxetine or paroxetine with BiteStrip assessment before treatment and on days 7 and 15	Masseter muscle activity increased after SSRI initiation, including among patients who did not report awareness of newly developed or worsening bruxism.	Only two SSRIs were evaluated over a short observation period, and BiteStrip does not provide full polysomnographic characterization.
Santos et al. [20]	Retrospective cross-sectional study of 583 patients attending a temporomandibular disorder clinic	Among 53 SSRI/SNRI users, 43 were identified as having bruxism; serotonergic antidepressant exposure was associated with greater odds of bruxism (OR, 2.52; 95% CI, 1.21-5.23).	SSRIs and SNRIs were analyzed together, the sample was drawn from a specialty-clinic population, and bruxism ascertainment was not based on standardized prospective physiologic assessment.
Montastruc [12]; Revet et al. [13]	International pharmacovigilance analyses	Identified reporting signals linking serotonergic antidepressants with bruxism and other movement disorders. One analysis found bruxism reporting to be more closely associated with norepinephrine transporter affinity than serotonin transporter affinity.	Spontaneous-reporting data cannot establish incidence or individual-level causality and are vulnerable to underreporting, reporting bias, incomplete clinical characterization, and absent exposure denominators.
Leonan-Silva et al. [21]	Critical appraisal of case reports, case series, and cross-sectional studies	Identified substantial heterogeneity in bruxism assessment; polysomnography was used in only one study, and awake and sleep bruxism were inconsistently differentiated.	The underlying literature remained heterogeneous and predominantly observational, limiting causal inference and comparison across studies.

Clinical implications

The recognition of SSRI-associated bruxism is clinically important because jaw clenching, tooth grinding, jaw pain, and related orofacial symptoms may overlap with primary dental conditions, temporomandibular disorders, anxiety-related muscle tension, or other forms of bruxism [8,21]. Evaluation should include the careful review of the temporal relationship between symptom onset and SSRI initiation or dose escalation, together with the consideration of whether symptoms occur during wakefulness, sleep, or both [8,21]. When the association is compelling, management may include dose reduction, discontinuation, or the substitution of the implicated antidepressant [8]. An early four-patient report by Ellison and Stanziani described nocturnal bruxism emerging within two to four weeks after the initiation of fluoxetine or sertraline, with remission after antidepressant dose reduction in one patient or the addition of buspirone in three [22]. Adjunctive pharmacologic approaches, including buspirone, have been reported in the literature, but the frequency of such reports should not be interpreted as evidence of comparative efficacy or as establishing a standard or first-line treatment [8,21].

The differential diagnosis should also include other medication-associated orofacial movement disorders, particularly oromandibular dystonia [13,23]. Bruxism is characterized by repetitive jaw muscle activity involving clenching, grinding, bracing, or thrusting [1,2], whereas oromandibular dystonia more typically involves sustained or patterned involuntary contractions affecting the jaw, tongue, or lower facial muscles, with phenotypes that may include abnormal jaw opening, closing, or deviation [23]. Clinical overlap is possible, particularly with jaw-closing dystonia; however, persistent abnormal jaw posturing, prominent jaw opening or deviation, or associated lingual or lower facial movements should raise the consideration of dystonia rather than bruxism alone [23].

Overall, the findings of this review support a cautious and individualized approach to SSRI-associated bruxism. The clinical association between SSRI exposure and bruxism is more firmly supported than any specific neuropharmacologic mechanism, while the evidence guiding treatment remains predominantly observational and case-based [8,21]. Management should therefore consider symptom severity, the strength of the temporal relationship with SSRI exposure, and the therapeutic value of maintaining an otherwise effective antidepressant. Clinical response to a particular intervention should likewise be interpreted as evidence of possible therapeutic benefit rather than confirmation of a specific pathogenic pathway.

Among the reported pharmacologic interventions, adjunctive buspirone has received the greatest clinical attention and is the most consistently described adjunctive pharmacologic strategy in the published case-based literature on SSRI-associated bruxism [8,10,11,16-18]. Reports involving several serotonergic antidepressants have described improvement following the addition of buspirone, including cases in which the implicated SSRI was continued [10,11,16-18]. These observations are clinically relevant because buspirone may provide a management option when dose reduction or the discontinuation of an otherwise effective SSRI is undesirable. However, the available evidence remains largely limited to case reports and small case series, and treatment response should not be interpreted as proof of efficacy or of a specific neuropharmacologic mechanism.

Reported buspirone regimens have varied across the case-based literature. In the systematic review by Garrett and Hawley, buspirone doses generally ranged from 5 to 30 mg/day administered in one to three divided doses, and symptom cessation was commonly reported within approximately two to three weeks after intervention [8]. In the paroxetine-associated case reported by Milanlioglu, buspirone was initiated at 5 mg nightly and increased to 10 mg nightly, with the disappearance of bruxism, jaw pain, and stiffness within two weeks [16]. Kohat and Chenchula more recently reported the complete resolution of persistent teeth grinding within four weeks after buspirone 5 mg/day was added [18]. Treatment duration and longer-term follow-up were inconsistently reported across cases, and these observations do not establish an optimal dose, dosing schedule, time to response, or the duration of therapy. Representative clinical and pharmacologic evidence concerning buspirone is summarized in Table 3.

**Table 3:** Representative clinical and pharmacologic evidence concerning buspirone in SSRI-associated bruxism This table summarizes representative clinical and pharmacologic evidence concerning buspirone as a potential management strategy for SSRI-associated bruxism. Unlike Table 1, which summarizes proposed pathophysiologic hypotheses, this table focuses on the evidence underlying the reported clinical use of buspirone. The available treatment literature is predominantly case-based, and no randomized controlled trials have established the efficacy, optimal dose, treatment duration, or comparative effectiveness of buspirone for SSRI-associated bruxism. Pharmacologic observations concerning 5-HT1A signaling provide mechanistic context but should not be interpreted as proof of the mechanism responsible for clinical improvement 5-HT1A, 5-hydroxytryptamine (serotonin) receptor 1A; SSRI, selective serotonin reuptake inhibitor

Evidence source	Evidence type	Principal observation	Interpretation and limitations
Garrett and Hawley [8]	Systematic review of published case reports	Adjunctive buspirone was among the repeatedly reported management strategies associated with improvement in antidepressant-associated bruxism.	Provides a synthesis of accumulated clinical experience but is based primarily on published case reports and is therefore vulnerable to publication bias, heterogeneous assessment, and the absence of controlled comparison.
Bostwick and Jaffee [10]	Four-case series	Improvement in SSRI-associated bruxism was reported following adjunctive buspirone, while antidepressant treatment was continued.	Provides direct clinical observations supporting buspirone as a potential adjunctive strategy, but the uncontrolled design cannot establish treatment efficacy or mechanism.
Sabuncuoglu et al. [11], Milanlioglu [16], Ak et al. [17], and Kohat and Chenchula [18]	Individual case reports and small case-based observations	Additional reports across different serotonergic antidepressant exposures described improvement following adjunctive buspirone.	Recurrence of similar treatment responses across reports strengthens the clinical signal, but small sample sizes, heterogeneous presentations, concomitant treatment changes, and selective reporting limit causal inference.
Liu et al. [14]	Experimental pharmacologic study	Acute and chronic buspirone administration produced differing 5-HT1A-dependent neurobehavioral and neurochemical effects.	Provides pharmacologic context for possible 5-HT1A-mediated actions of buspirone but did not investigate bruxism and did not establish the restoration of dopaminergic neurotransmission as the therapeutic mechanism.

The pharmacologic properties of buspirone provide additional mechanistic context but should be interpreted cautiously. Buspirone has partial agonist activity at 5-HT1A receptors, and experimental evidence indicates that its neurobehavioral and neurochemical effects differ between acute and chronic administration and are partly dependent on 5-HT1A signaling [10,11,14]. These observations are compatible with broader downstream monoaminergic modulation rather than a single established receptor-level mechanism. Accordingly, clinical response to buspirone does not demonstrate that 5-HT1A dysfunction is present or causative, nor does it establish that the restoration of dopaminergic signaling mediates improvement [10,11,14].

The evidence supporting treatment remains limited. No randomized controlled trials have established the efficacy of buspirone, optimal dose, time to response, treatment duration, comparative effectiveness, the durability of benefit, or predictors of response [21]. Buspirone therefore should not be regarded as an established standard or first-line treatment, although it remains the most consistently described adjunctive pharmacologic strategy in the published case-based literature. Future prospective studies should compare adjunctive buspirone with SSRI dose reduction, discontinuation, antidepressant substitution, and other management approaches using standardized measures of bruxism activity and clinically meaningful outcomes.

Strengths, limitations, and future directions

This review has several strengths. It integrates clinical case-based evidence, observational studies, instrument-based findings, pharmacovigilance analyses, and relevant neuropharmacologic literature within a focused framework for SSRI-associated bruxism. Particular emphasis was placed on distinguishing SSRI-specific evidence from broader antidepressant-associated findings, separating clinical association from mechanistic inference, and differentiating awake and sleep bruxism when the underlying studies permitted. The review also evaluates proposed mechanisms, alongside their evidentiary limitations, and separately summarizes the overall clinical evidence and the case-based literature concerning buspirone. This approach provides a clinically oriented synthesis while avoiding stronger causal or therapeutic conclusions than the available evidence supports.

Several limitations of the current evidence base restrict definitive conclusions regarding the incidence, pathophysiology, and optimal management of SSRI-associated bruxism. Much of the literature consists of case reports, case series, observational studies, pharmacovigilance analyses, and reviews of heterogeneous clinical reports [8,21]. Although these sources demonstrate recurring temporal associations between SSRI exposure and bruxism, interpretation is limited by publication bias, incomplete clinical characterization, confounding, and the inconsistent ascertainment of both medication exposure and bruxism outcomes. The available evidence therefore supports a clinically meaningful association more strongly than it establishes incidence, comparative risk among individual SSRIs, or causality in individual patients.

A major limitation is the heterogeneity of bruxism assessment. Many reports rely on patient-described jaw clenching or tooth grinding or on routine clinical observation, while awake and sleep bruxism are inconsistently distinguished and objective physiologic assessment remains uncommon [21]. Self-reported symptoms, clinically identified bruxism, portable electromyographic measurements, and polysomnographically characterized sleep bruxism represent related but noninterchangeable outcomes. Future prospective studies should apply standardized definitions, clearly distinguish awake from sleep manifestations, and incorporate quantitative jaw muscle activity or polysomnographic assessment when appropriate. Ideally, bruxism should be characterized before and after SSRI initiation or dose modification to distinguish treatment-emergent changes from preexisting behavior and better define temporal and dose-related relationships.

Mechanistic interpretation remains similarly constrained. The serotonin-dopamine imbalance hypothesis provides a biologically plausible framework, but direct evidence demonstrating reduced dopaminergic neurotransmission or a specific receptor-level abnormality in affected patients is lacking [8,10]. Pharmacovigilance findings suggesting possible noradrenergic involvement and experimental evidence concerning 5-HT1A signaling further indicate that SSRI-associated bruxism is unlikely to reflect the dysfunction of a single neurotransmitter pathway [12-14]. Future mechanistic studies should therefore examine interactions among serotonergic, dopaminergic, noradrenergic, and distributed motor-control systems rather than testing a serotonin-only model. Interindividual variation in SSRI exposure represents an additional testable susceptibility hypothesis. CYP2D6 genotype influences paroxetine metabolism, whereas CYP2C19 genotype influences the metabolism of citalopram, escitalopram, and sertraline, and genotype-based prescribing recommendations are available for these agents [24]. Fluoxetine is also metabolized through CYP2D6-dependent pathways, although its pharmacokinetics are more complex and current CPIC guidance does not provide a genotype-based dosing recommendation for fluoxetine [24]. Reduced metabolic capacity or clinically relevant drug interactions could plausibly increase SSRI exposure at a given dose and thereby contribute to interindividual susceptibility or symptom emergence following dose escalation. However, CYP genotype, altered metabolic capacity, drug interactions affecting exposure, and measured SSRI concentrations have not been directly evaluated as predictors of SSRI-associated bruxism. These factors should therefore be regarded as specific, testable future research directions rather than established risk factors.

Treatment evidence is particularly limited. Although adjunctive buspirone is the most consistently described pharmacologic strategy and has been associated with improvement despite continued SSRI exposure, the evidence remains predominantly case-based [8,10,11,16-18]. No randomized controlled trials have established its efficacy, optimal dose, time to response, treatment duration, durability of benefit, recurrence after discontinuation, tolerability, or predictors of response [21]. It is also unknown whether treatment response differs according to the implicated SSRI or the clinical phenotype of bruxism. Future prospective studies should therefore characterize buspirone-specific treatment parameters and directly compare adjunctive buspirone with SSRI dose reduction, discontinuation, antidepressant substitution, and other management approaches. Standardized outcome measures incorporating both bruxism activity and clinically meaningful consequences such as pain, dental injury, sleep disruption, and functional impairment would help determine whether reported symptomatic improvement translates into reproducible clinical benefit.

Future research should prioritize prospective longitudinal studies with clearly characterized SSRI exposure, the standardized assessment of awake and sleep bruxism, and systematic evaluation before and after treatment initiation or dose changes. Studies should also examine individual-drug differences, dose-response relationships, pharmacokinetic and pharmacogenetic susceptibility factors, and relationships between objective motor activity and patient-important outcomes. Mechanistic and therapeutic investigations should remain distinct so that clinical response to an intervention such as buspirone is not interpreted as proof of a specific pathogenic pathway. A more rigorous characterization of both the clinical phenotype and treatment response will be necessary to clarify causality, identify patients at increased risk, and establish evidence-based strategies that allow effective antidepressant therapy to be maintained whenever possible.

## Conclusions

SSRI-associated bruxism is a clinically meaningful medication-associated phenomenon characterized by recurring temporal relationships with antidepressant initiation or dose escalation and improvement after medication modification in many reported cases. Proposed mechanisms are likely multifactorial and may involve altered monoaminergic regulation within central motor networks, including serotonergic-dopaminergic interactions, 5-HT1A signaling, and possible noradrenergic contributions; however, these mechanisms remain unconfirmed in affected patients.

Adjunctive buspirone is the most consistently described pharmacologic strategy and may allow the continuation of an otherwise effective SSRI, although its apparent benefit remains supported primarily by case-based evidence and should not be interpreted as proof of a specific neuropharmacologic mechanism. More rigorous prospective studies using standardized bruxism definitions, a clearer differentiation of awake and sleep manifestations, and objective physiologic assessment are needed to clarify incidence, susceptibility, mechanisms, and treatment response. The better characterization of this association may improve clinical recognition and support management strategies that preserve effective antidepressant therapy whenever possible.
